# Immunometabolic Changes in Glia – A Potential Role in the Pathophysiology of Obesity and Diabetes

**DOI:** 10.1016/j.neuroscience.2019.10.021

**Published:** 2020-11-01

**Authors:** Josephine L. Robb, Nicole A. Morrissey, Paul G. Weightman Potter, Hannah E. Smithers, Craig Beall, Kate L.J. Ellacott

**Affiliations:** Neuroendocrine Research Group, Institute of Biomedical & Clinical Sciences, University of Exeter Medical School, Exeter, UK

**Keywords:** AraC, arabinofuranosyl cytidine, ARC, arcuate, DMH, dorsomedial hypothalamus, GFAP, glial-fibrillary acidic protein, HFHS, high-fat high-sucrose, IGF-1, insulin-like growth factor 1, IKK, IκB kinase, LPS, lipopolysaccharide, NF-κB, nuclear factor-kappa B, OVLT, organum vasculosum of the lamina terminalis, PI3K, PI3-kinase, PVN, paraventricular nucleus of the hypothalamus, STAT3, signal transducer and activator of transcription 3, TLRs, toll-like receptors, VMH, ventromedial nucleus, astrocyte, microglia, inflammation, obesity, diabetes, immunometabolism

## Abstract

Chronic low-grade inflammation is a feature of the pathophysiology of obesity and diabetes in the CNS as well as peripheral tissues. Glial cells are critical mediators of the response to inflammation in the brain. Key features of glia include their metabolic flexibility, sensitivity to changes in the CNS microenvironment, and ability to rapidly adapt their function accordingly. They are specialised cells which cooperate to promote and preserve neuronal health, playing important roles in regulating the activity of neuronal networks across the brain during different life stages. Increasing evidence points to a role of glia, most notably astrocytes and microglia, in the systemic regulation of energy and glucose homeostasis in the course of normal physiological control and during disease. Inflammation is an energetically expensive process that requires adaptive changes in cellular metabolism and, in turn, metabolic intermediates can also have immunomodulatory actions. Such “immunometabolic” changes in peripheral immune cells have been implicated in contributing to disease pathology in obesity and diabetes. This review will discuss the evidence for a role of immunometabolic changes in glial cells in the systemic regulation of energy and glucose homeostasis, and how this changes in the context of obesity and diabetes.

## Highlights

• Glia direct respond to changes in nutrients and hormones regulating energy balance.
• Microglia and astrocytes are implicated in the pathophysiology of obesity and diabetes.
• Obesity causes gliosis: reversible changes in glia structure and function.
• In metabolic disease inflammation-driven changes in glial likely regulates neurons.
• Glial regulation of neural circuits controlling energy balance requires more study.

## Graphical abstract

**Figure:**
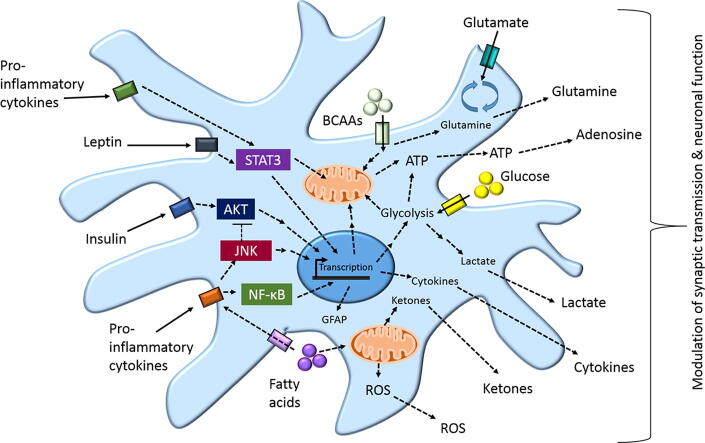

## Introduction

Inflammation in the brain is a key feature of disease pathology seen in experimental models of obesity and diabetes (De Souza et al., 2005, Thaler et al., 2012). Evidence from human brain imaging studies also supports the existence of obesity-associated neuroinflammation in both adults and children (Thaler et al., 2012, Schur et al., 2015, Berkseth et al., 2018, Sewaybricker et al., 2019).

In the brain, neuroinflammation is mediated in part by a class of non-neuronal cells collectively known as glia. Glia are specialised cells which cooperate to promote and preserve neuronal health, playing important roles in regulating the activity of neuronal networks across the brain during different life stages. A key feature of glia is their sensitivity to changes in the CNS microenvironment and the ability to rapidly adapt their function accordingly (Wolf et al., 2017, Verkhratsky and Nedergaard, 2018). For example, when the CNS is damaged or during disease, glia exhibit reactive gliosis characterised by rapid adaptive changes in form and function aimed at minimising neuronal damage and restoring tissue homeostasis (Burda and Sofroniew, 2014). When present chronically, reactive gliosis can contribute to disease pathology. Throughout this review the term ‘gliosis’ will be used to refer to reactive glial changes in the CNS, although we acknowledge that glia in the peripheral and enteric nervous systems are also likely to show changes in obesity and diabetes.

The aim of this article is to present an overview of the evidence for the contribution of the principal immune competent glia, namely astrocytes and microglia, in the pathology of obesity and diabetes, focusing on the emerging evidence for a role of metabolic changes in fuelling their plasticity and inflammatory responses. Other glia including tanycytes and oligodendrocytes (notably NG2 glia) have also been studied to a lesser extent in this context, but will not be highlighted here. The recent review by Verkrahsky and Nedergaard is an excellent resource for those seeking comprehensive information on the cellular physiology of astrocytes (Verkhratsky and Nedergaard, 2018), while readers are referred to the article of Wolf and colleagues for a similarly extensive review of microglial physiology (Wolf et al., 2017).

### Immunometabolism: inflammation and metabolism are intrinsically linked

The inflammatory response is an energetically expensive process requiring the synthesis and secretion of new proteins and in some cases, changes in cellular motility and morphology. In peripheral immune cells, a diverse range of metabolic changes essential for their function have been characterised (O’Neill et al., 2016). Although the exact nature of these “immunometabolic” changes varies according to the cell type examined, they have been seen consistently across numerous studies. Chronic exposure to inflammatory stimuli results in metabolic reprogramming of peripheral immune cells, and emerging evidence suggests that metabolic intermediates such as citrate (Williams and O’Neill, 2018) and itaconate (Hooftman and O’Neill, 2019) can themselves have immunomodulatory actions.

Obesity and diabetes result in immunometabolic changes in peripheral immune cells which have been implicated in disease pathology (Hotamisligil, 2017). Indeed, metformin which is a first line therapeutic agent for type-2 diabetes has anti-inflammatory effects (Vasamsetti et al., 2015, Cameron et al., 2016), some of which are linked to a role in modulating cellular metabolism (Vasamsetti et al., 2015). This has resulted in investigation of the therapeutic efficacy of metformin in autoimmune conditions including lupus and multiple sclerosis (Norata et al., 2015).

There is developing evidence that immunometabolic changes are also seen in glia (most notably microglia (Paolicelli and Angiari, 2019) and astrocytes (Vicente-Gutierrez et al., 2019)). Although not yet as comprehensively studied as in peripheral immune cells, these are potential therapeutic targets for neurological disease.

## Aetiology of gliosis in obesity and diabetes

When considering the possible role for immunometabolic changes in glia in the pathology of obesity and diabetes, it is important to first discuss the potential causes of gliosis in metabolic disease. Although most of the studies mentioned in this section examined animal models of obesity/type-2 diabetes, there is evidence of CNS gliosis in streptozotocin (STZ)-induced rodent models of type-1 diabetes (Coleman et al., 2004). However, it is less clear whether this gliosis is related to the proinflammatory nature of the STZ treatment and/or the dysregulation of glycaemic control seen in these animals.

### Nutrient composition of the diet

Markers of both microgliosis and astrogliosis have been observed within the hypothalamus of obese animals and humans (Horvath et al., 2010, García-Cáceres et al., 2011, Fuente-Martín et al., 2012, Thaler et al., 2012, Buckman et al., 2013, Berkseth et al., 2014, Gao et al., 2014, Schur et al., 2015, Berkseth et al., 2018). Extra-hypothalamic changes have also been observed (Pistell et al., 2010, Buckman et al., 2013), with the hippocampus being the most studied brain region in this regard. It remains unclear whether the effects of obesity on hypothalamic gliosis are dependent or independent of diet. Several studies observe hypothalamic gliosis in genetic mouse models of obesity, characterised by hyperphagia on a standard chow diet. This gliosis is independent of the animals eating an obesogenic high-fat high-sucrose (HFHS) diet (Buckman et al., 2013, Gao et al., 2014) and may be related to hormonal alterations in these animals (Gao et al., 2014). In wild-type mice, oral gavage of saturated fatty acids is sufficient to induce microgliosis but not astrogliosis in the medial basal hypothalamus (Valdearcos et al., 2014), resembling microgliosis seen following consumption of a HFHS diet. However, an independent study suggests that it is the combination of high-fat and high-sucrose in the diet that is an important trigger for the development of hypothalamic gliosis, as prolonged consumption of a high-fat low-carbohydrate diet did not have the same proinflammatory effects (Gao et al., 2017a). As such, while several independent groups have demonstrated an effect of obesity on hypothalamic gliosis, the contribution of specific dietary nutrients in this process is still unclear.

In mice, diet-induced obesity-associated changes in hypothalamic astrogliosis appear to be reversible following weight-loss caused by returning animals to a standard chow diet or following bariatric surgery (Berkseth et al., 2014, Herrick et al., 2018). Imaging studies in humans also indicate improvements in markers of hypothalamic inflammation following bariatric surgery-induced weight loss (van de Sande-Lee et al., 2019). It is unclear whether after weight-loss hypothalamic glia become ‘primed’ to subsequent inflammatory insults, or have an altered function which may contribute to the susceptibility to rebound weight-gain seen in individuals following weight loss. Indeed, in rodents, *in utero* or neonatal exposure to a HFHS diet is associated with microgliosis, microglial priming to subsequent inflammatory insults, and increased susceptibility to the development of diet-induced obesity in adulthood (Bilbo and Tsang, 2010, Ziko et al., 2014).

The age at which mice are placed on an obesogenic diet appears to be a critical determining factor in the development of diet-induced hypothalamic gliosis. Mice which are placed on an obesogenic HFHS diet as juveniles appear to be relatively resistant to the development of hypothalamic gliosis, compared with animals who start the diet later in life (Freire-Regatillo et al., 2019). These differences may be caused by region specific age-related changes in glial function, as functional and transcriptomics studies point to differential activity of metabolic pathways in astrocytes from young compared with older mice (Clarke et al., 2018, Santos et al., 2018). It is possible that differences in the age at which animals were placed on obesogenic diets may explain the discordance in the results between some published studies looking at obesity-associated gliosis.

### Peripheral inflammation and changes in the gut microbiome

Obesity is associated with chronic peripheral low-grade tissue inflammation, which likely contributes to the increased susceptibility of obese individuals to developing comorbidities, including cardiovascular and cerebrovascular disease, cancer, and diabetes. Changes in the immunometabolic profile of peripheral immune cells have been reported in animals and people with obesity and type-2 diabetes (Hotamisligil, 2017). The gut microbiome is also altered in people with obesity and is believed to contribute to the changes in peripheral inflammatory state (Torres-Fuentes et al., 2017).

Whether peripheral inflammatory changes contribute to CNS inflammation and gliosis in obesity is unclear, but it is likely to be a contributing factor. Hypothalamic vascularization and permeability of the blood brain barrier are increased in obese rodents (Yi et al., 2012), and select inflammatory cytokines have been shown to cross the blood brain barrier in high enough concentrations to affect the function of both the barrier and the brain itself (Pan et al., 2011). As the hypothalamus contains two circumventricular sites, where the blood brain barrier is more permeable to entrance of substances from the periphery, namely the organum vasculosum of the lamina terminalis (OVLT) and the median eminence, it is probable that the function of at least the ventral portions of the hypothalamus are thus impacted. The microbiota-gut-brain axis may modulate CNS function, either directly via activity of the vagus nerves or indirectly by altering peripheral inflammation or modulating neuroendocrine activity (Torres-Fuentes et al., 2017).

There is evidence from two independent research groups, including our own, that in mice obesity is associated with increased migration of peripheral immune cells into the CNS where they phenotypically resemble microglia (Buckman et al., 2014, Valdearcos et al., 2017). Thus, a contribution of these cells to obesity-associated hypothalamic inflammation/gliosis cannot be ruled out.

Hypothalamic glial changes are evident within 24 h of rodents being switched from their standard diet to an obesogenic HFHS diet (Thaler et al., 2012, Buckman et al., 2015). This is prior to the onset of chronic white adipose tissue and liver inflammation associated with obesity. This suggests that, at least acutely, hypothalamic glia are directly responsive to the dietary change. However, the contribution of an acute transient peripheral inflammatory response to a HFHS meal cannot be ruled out.

### Neurogenic neuroinflammation

In the hypothalamus, obesity-associated astrogliosis in adult mice is not uniform. Some hypothalamic nuclei, such as the dorsomedial hypothalamus (DMH) and the arcuate (ARC), show profound astrogliosis while other nuclei, such as the ventromedial nucleus (VMH), are relatively spared (Buckman et al., 2013). Similarly, regional differences in obesity-associated hypothalamic microgliosis have also been reported, with the ARC and median eminence area being impacted, while the VMH is again relatively spared (Valdearcos et al., 2017). As the DMH is not adjacent to a circumventricular site it is probable that the astrogliosis in this region is not directly caused solely by peripheral inflammatory changes or constituents of the diet, such as saturated fatty acids, which may modulate the activity of glia. One possible explanation is functional differences between glial cells within the hypothalamus, as regional heterogeneity in glial cell function in the brain is an emerging area of understanding (Cali et al., 2019, Masuda et al., 2019, Polyzos et al., 2019). Another likely factor is ‘neurogenic neuroinflammation’ (Xanthos and Sandkuhler, 2014). Increased activity of neurons, particularly in response to an intensive or traumatic stimulus, causes neuronal metabolic stress which necessitates increased activity of the local glial cells that help sustain neuronal activity by supplying metabolic support, mediating neurotransmitter recycling, and maintaining overall tissue homeostasis (Wolf et al., 2017, Verkhratsky and Nedergaard, 2018). As such, increased activity of neurons likely results in a concomitant increase in activity of glia in the immediate vicinity.

Further support for neurogenic neuroinflammation as a likely contributor to obesity-associated gliosis comes from studies that have examined glial changes following nutrient insufficiency. In common with obesity, states of low systemic energy availability in mice, including fasting (Fuente-Martín et al., 2012, Zhang et al., 2017), caloric restriction (Harrison et al., 2019), and hypoglycaemia (McDougal et al., 2013), induce changes in astrocyte morphology and functional markers indicative of astrogliosis in the brain, in regions where increased neuronal activity has been reported in these conditions.

In summary, obesity-associated gliosis has been observed by several independent research groups in different experimental models. The causes of this gliosis are not yet fully elucidated but are likely to be multi-faceted, encompassing responses to changes within the brain and the periphery.

## Glial inflammatory signalling is implicated the pathology of obesity & diabetes

### Nuclear factor-kappa B (NF-κB) signalling

The nuclear factor-kappa B (NF-κB) family of transcription factors regulate the expression of genes related to cytokine production and cell survival in inflammatory states (Liu et al., 2017). During non-inflammatory states NF-κB activity is inhibited by the IκB proteins. Signalling pathways regulating NF-κB activity are controlled by a variety of upstream receptors, including Toll-Like Receptors (TLRs), cytokine receptors and growth factor receptors. Activation of signalling pathways downstream of these receptors leads to activation of IKK-β, the catalytic subunit of the IκB kinase (IKK) complex, which phosphorylates IκB proteins. This results in degradation of IκB, allowing phosphorylation and translocation of NF-κB family members (p50, p52, p65, RelB and c-Rel) to the nucleus where, when bound as homo- or heterodimers, they act as transcription factors modulating gene expression. This is termed the canonical NF-κB pathway. Alternatively, NF-κB activity can be triggered through the non-canonical pathway by processing of the C-terminal structure of p52 precursor protein, p100, resulting in translocation to the nucleus (Liu et al., 2017). For a review of the functions of NF-κB signalling in different CNS cell types, the reader is referred to the recent article by Dresselhaus and Meffert (Dresselhaus and Meffert, 2019).

### Modulation of nuclear factor-kappa B (NF-κB) signalling in astrocytes impacts systemic energy homeostasis

Proteomic profiling of the astrocytic response to proinflammatory cytokines and the bacterial coat protein lipopolysaccharide (LPS) suggests that these stimuli differentially activate the canonical and non-canonical NF-κB signalling pathways (Dozio and Sanchez, 2018). In addition to genes involved in inflammatory signalling and cell survival, the structural protein vimentin (Zheng et al., 2005) is an NF-κB target gene; thus, potentially linking changes in inflammatory signalling and morphology in astrocytes. Astrocytic ensheathment of synapses has been implicated in the modulation of the activity of hypothalamic melanocortin neuronal circuits regulating feeding behaviour (Horvath et al., 2010), representing one mechanism by which changes in astrocyte activity may impact systemic energy homeostasis.

Genetically mediated inducible-inhibition of NF-κB signalling in glial-fibrillary acidic protein (GFAP)-expressing cells (which are predominantly astrocytes), via expression of a form of IκB that cannot be targeted for degradation by phosphorylation, prevents acute hypothalamic astrogliosis in mice fed HFHS-diet for 24 h (Buckman et al., 2015). This suggests that NF-κB signalling in astrocytes is responsible, at least in part, for the acute responsiveness of hypothalamic astrocytes to a HFHS diet. This study, performed by our group, found that inhibiting the acute HFHS diet-induced hypothalamic astrogliosis in mice results in increased food intake, but only during the acute hyperphagic response to the diet (first 24 h), suggesting that activation of NF-κB signalling in astrocytes may be part of a homeostatic response to deviations from energy homeostasis (Buckman et al., 2015).

In addition to impacting the acute physiological response to a HFHS diet, genetic modulation of NF-κB signalling in astrocytes changes energy homeostasis in mice chronically consuming this obesogenic diet. Genetically mediated inducible-deletion of IKKβ in GFAP-expressing cells reduces hypothalamic astrogliosis in mice previously fed a HFHS diet for 6 weeks (Douglass et al., 2017). Moreover, this leads to reduced food intake, attenuation of further weight gain and improved glucose homeostasis in these animals. In mice, germ-line genetic modification of IKKβ activity in GFAP-expressing cells provides further support for a key role of this pathway in astrocytic regulation of energy homeostasis: increased constitutive activity of IKKβ in GFAP expressing cells increases food intake and weight gain in response to a HFHS diet, while partial ablation of IKKβ activity in these cells shows the opposite effect (Zhang et al., 2017).

### Modulation of nuclear factor-kappa B (NF-κB) signalling in microglia impacts systemic energy homeostasis

In common with astrocytes, NF-κB signalling in microglia can also play a role in regulating energy homeostasis. Depletion of CNS resident microglia in mice using the drug PLX5622 reduces food intake and body weight gain in animals fed a HFHS diet, but not standard chow (Valdearcos et al., 2017). In a related study, administration of the anti-mitotic drug arabinofuranosyl cytidine (AraC) into the brain also blunts food intake and weight gain on this diet (Andre et al., 2017). The underlying mechanism is thought to be through preventing HFHS-induced cell-proliferation, likely encompassing both microglia and astrocytes. Together, these studies indicate that microglia likely have a regulatory role in the pathophysiological response to an obesogenic diet. Genetically mediated inducible-deletion of microglial IKKβ diminishes HFHS-induced microgliosis, which is accompanied by reduced weight-gain and food intake, indicating relative protection from some obesogenic effects of the diet (Valdearcos et al., 2017). In contrast, the same study revealed that increasing inflammatory activity in microglia mimics the hypothalamic microgliosis and weight-gain associated with HFHS feeding (Valdearcos et al., 2017).

Together, these studies indicate that NF-κB signalling is a key regulatory node in glial function, the modulation of which impacts systemic energy homeostasis.

### Signal transducer and activator of transcription 3 (STAT3) signalling

Signal transducer and activator of transcription 3 (STAT3) is an important signalling molecule contributing to normal glial function (Ceyzeriat et al., 2016). Activation of STAT3 signalling, following tyrosine phosphorylation by members of the Janus kinase family (JAK; Tyr^705^) or serine phosphorylation by a variety of kinases including ERK or JNK MAP kinase (Ser^727^), has several potential downstream consequences. The classical pathway, following JAK mediated phosphorylation of Tyr^705^, results in dimerization of STAT3 and translocation to the nucleus where it mediates gene transcription. The non-classical pathway, kinase mediated phosphorylation of Ser^727^, promotes STAT3 recruitment to the mitochondrion leading to regulation of cellular metabolism (Yang and Rincon, 2016). STAT3 signalling is downstream of receptors for cytokines and the hormone leptin.

### Glial STAT3 signalling is implicated in the regulation of food intake and body weight

Several studies implicate STAT3 signalling in astrocytes in the regulation of systemic energy homeostasis. Leptin receptors (Ob-Rb) are expressed on astrocytes (Pan et al., 2008, Hsuchou et al., 2009) and leptin has been demonstrated to impact astrocyte function *in vitro* and *in vivo* (Hsuchou et al., 2009, García-Cáceres et al., 2011, Fuente-Martín et al., 2012). Genetically mediated germ-line deletion of leptin receptors from GFAP-expressing cells in mice attenuates the inhibitory effects of exogenously administered leptin on food intake and results in enhanced fast-induced refeeding. This suggests that leptin-signalling in astrocytes plays a role in regulating neural circuits controlling feeding (Kim et al., 2014). This is supported by evidence indicating changes in astrocyte morphology and synaptic input to hypothalamic melanocortin neurons in these animals (Kim et al., 2014). There is also evidence to suggest that loss of leptin receptor signalling in astrocytes impacts the pathophysiological response to diet-induced obesity; however, the phenotype appears to be complex and variable depending on the genetic-targeting approach taken, so further clarification is required (Jayaram et al., 2013, Wang et al., 2015).

As well as astrocytes, leptin receptors are also expressed on microglia, with leptin treatment modulating microglial morphology and stimulating cytokine production and release (Pinteaux et al., 2007, Lafrance et al., 2010). Genetically mediated germ-line deletion of leptin receptors from myeloid cells, including microglia, results in enhanced weight gain on a HFHS diet associated with attenuation of diet-induced microgliosis in the paraventricular nucleus of the hypothalamus (PVN) and alterations in the ARC-PVN melanocortin circuit controlling feeding (Gao et al., 2018). This suggests a role for microglial leptin signalling in regulating neural circuits controlling feeding.

In addition to activation by leptin, STAT3 can be phosphorylated through signal transduction downstream of the interleukin (IL)-6 receptor (IL-6R). IL-6 is an important pleiotropic cytokine implicated in the central regulation of energy and glucose homeostasis (Wallenius et al., 2002, Timper et al., 2017, Mishra et al., 2019). IL-6 and its receptor are produced and expressed by both astrocytes and microglia (Erta et al., 2012). As such, IL-6 (neuronal or glial in origin) may act in a paracrine or autocrine manner to impact glial function. Genetically mediated germ-line deletion of IL-6, but not IL-6R, from GFAP-expressing cells results in a modest increase in body weight (standard chow diet) in male but not female mice (Quintana et al., 2013) suggesting a potential role of astrocyte derived IL-6 in the regulation of energy homeostasis.

### AKT signalling

AKT signalling is a pivotal regulator of cellular pathways throughout the body, implicated in functions from survival and proliferation to metabolism and growth (Manning and Toker, 2017). In the context of obesity and diabetes perhaps the best understood functions of AKT signalling are those downstream of insulin receptor activation. Insulin receptors are expressed in the brain and the action of insulin in the CNS regulates numerous functions; although, how insulin enters the brain to exert its actions is a matter of debate (Gray et al., 2014). When insulin receptors bind insulin, a signalling pathway is initiated by a conformational change to the insulin receptor. This leads to phosphorylation of PI3-kinase (PI3K) and subsequent phosphorylation of AKT. Downstream of insulin receptor activation, AKT signalling regulates glucose and glutamate transporter expression, glucose storage as glycogen, and protein synthesis via activation of mTOR (Manning and Toker, 2017).

### Modulation of astrocytic AKT transduction impacts cellular metabolism and systemic glucose homeostasis in mice

Within the CNS astrocytes are cellular targets of insulin action. Human astrocytes in culture express functional insulin receptors, and inhibition of PI3K-AKT signalling with LY294002 reduces the insulin-mediated increases in glycogen synthesis and storage in these cells, indicating that this pathway is important for regulating CNS glucose availability (Heni et al., 2011). Recent work suggests that cooperative action of insulin and insulin-like growth factor 1 (IGF-1) promotes astrocyte glucose uptake and may be important for the neural response to hypoglycaemia (Fernandez et al., 2017). In mouse astrocytes, loss of insulin receptor signalling also impacts cellular glucose metabolism, mitochondrial function, and ATP production (Garcia-Caceres et al., 2016, Cai et al., 2018).

With respect to systemic energy homeostasis, genetically mediated inducible-deletion of insulin receptors from astrocytes increases food intake and reduces glucose tolerance in mice, an effect that is recapitulated when deletion of astrocyte insulin receptors is restricted to the hypothalamus (Garcia-Caceres et al., 2016). Interestingly, in an independent study, genetically mediated germ-line deletion of insulin receptors from mouse astrocytes replicated the mild-worsening of glucose tolerance, while the effect on food intake was no longer present (Cai et al., 2018). However, these animals did demonstrate a depressive-like phenotype which was attributed to alterations in dopamine neuronal activity in the mesolimbic system (Cai et al., 2018).

Genetically mediated inducible-deletion of astrocyte insulin receptors alters astrocyte morphology, which likely impacts the activity of the hypothalamic melanocortin circuitry contributing to the effects on systemic energy homeostasis (Garcia-Caceres et al., 2016). Evidence suggests that AKT signalling is also important for regulating expression of glutamate transporter-1 (GLT-1/excitatory amino acid transporter 2 [EAAT2]), and thus glutamate cycling in astrocytes (Li et al., 2006, Wu et al., 2010), which may represent another mechanism by which loss of insulin signalling in adult astrocytes could impact hypothalamic neuronal activity. Importantly, the work of García-Cáceres and colleagues also implicates astrocytes in the transport of insulin across the blood brain barrier (Garcia-Caceres et al., 2016).

### c-Jun NH_2_-terminal kinase (JNK) signalling

c-Jun NH_2_-terminal kinases (JNK) are a family of mitogen activated protein kinases (MAPKs) which are activated by multiple stimuli, including pro-inflammatory cytokines (Zeke et al., 2016). JNKs are activated through phosphorylation by several MAPK kinases, including MKK4 and MKK7 (Tournier et al., 2001, Johnson and Nakamura, 2007). There are three different isoforms of JNK (1–3) the functions of which are differentially implicated in obesity and insulin resistance: JNK1 and 2 activity promoting disease pathogenesis while JNK3 activity appears to be protective (Solinas and Becattini, 2017). When considering data reporting on JNK signalling it is important to be mindful of the fact that MKK4 also phosphorylates p38 MAPK, resulting in potential crosstalk between the JNK and p38 MAPK pathways (Zeke et al., 2016).

### JNK signalling regulates astrogliosis

JNK phosphorylation is associated with increased GFAP expression in the context of astrogliosis (Cole-Edwards et al., 2006, Tang et al., 2006, Gadea et al., 2008), mediated in part via AP-1 transcription factor binding to the GFAP promotor (Gao et al., 2013). This process is dependent on Ca^2+^ signalling as the Ca^2+^ chelator BAPTA-AM reduces both JNK phosphorylation and GFAP accumulation during *in vitro* scratch assays in mouse primary astrocytes (Gao et al., 2013). In addition to regulating GFAP expression, JNK signalling plays a role in regulating inflammation in astrocytes. For example, inhibition of JNK signalling in primary mouse astrocyte cultures with SP600125 reduces COX-2, IL-6 and iNOS expression in response to activation by multiple cytokines, including IL-1β, TNF-α and IFN-γ, despite activation NF-κB signalling remaining (Falsig et al., 2004). Together these studies indicate that JNK signalling plays an integral role in astrocyte function during astrogliosis and modulation of this pathway in glia has been explored as therapeutic target for neurological disease (Kaminska et al., 2009).

### Role of astrocyte JNK signalling in mediating insulin resistance

Although roles for JNK1 signalling in mediating insulin resistance through inhibition of insulin receptor signalling is well established in the periphery (Solinas and Becattini, 2017) and CNS (Belgardt et al., 2010), as yet there is limited direct data available examining the role of JNK signalling in glia in the context of obesity insulin resistance. Diet-induced obesity increases hypothalamic JNK activity and germ-line deletion of JNK1 from nestin expressing cells results in protection from diet-induced glucose intolerance and insulin resistance (Belgardt et al., 2010). As the intermediate filament protein nestin is expressed in neural stem cells and reactive astrocytes (which are a feature of diet-induced obesity in mice) a contribution of changes in JNK1 signalling in astrocytes to the phenotype seen cannot be ruled out; particularly considering the importance of this signalling pathway in regulating astrogliosis (described above).

### Links between glial inflammatory signalling and regulation of cellular metabolism

The specific mechanism(s) by which CNS inflammatory signalling is activated in glia in obese and/or diabetic states is unclear. Candidates include direct action of dietary nutrients (such as saturated fatty acids, as discussed elsewhere in this review) and cytokines from endocrine (peripheral tissues/cells), paracrine (neurons, endothelial cells, pericytes, and neighbouring glia) and/or autocrine (glia) sources. Diet-induced obesity leads to an increase in hypothalamic proinflammatory cytokine expression which is likely to be both glial and neuronal in origin (De Souza et al., 2005, Thaler et al., 2012). Genetically mediated microglial depletion attenuates the inflammatory response to both saturated fatty acids and LPS in mouse hypothalamic slice cultures (Valdearcos et al., 2014), indicating that microglia are a primary mediator of the hypothalamic inflammatory response to these stimuli.

In common with what has been observed in peripheral tissues, cytokines and other inflammatory stimuli (e.g. LPS) modulate cellular metabolism in glia (Yu et al., 1995, Gavillet et al., 2008, Belanger et al., 2011, Gimeno-Bayon et al., 2014, Nair et al., 2019). For example, *in vitro* treatment of primary mouse cortical astrocytes with the cytokines TNF-α and IL-1β promotes uptake of the glucose mimetic 2-deoxyglucose (2DG) and reduces glycogen stores (Yu et al., 1995, Gavillet et al., 2008, Belanger et al., 2011). In glia, given that NF-κB (Mauro et al., 2011), STAT3 (Demaria et al., 2010, Sarafian et al., 2010), AKT (Pousset et al., 2000, Garcia-Caceres et al., 2016, Lee et al., 2017, Cai et al., 2018) and JNK signalling have been independently implicated in regulating both the response to cytokines and cellular metabolism, it is likely that a localised inflammatory microenvironment in the hypothalamus (and potentially other brain regions) leads to glial immunometabolic changes that modify the function of appetite regulating neural circuits in this region. The potential role of glial derived metabolic intermediates in the regulation of these circuits is discussed in more detail below.

## Metabolic changes in glia are implicated in modifying behaviour

Glia are metabolically flexible cells which enables them to be exquisitely sensitive to local changes in the CNS microenvironment. Astrocytes can also store glycogen for use as a fuel reserve under conditions of low energy availability (Brown et al., 2005, Matsui et al., 2012). This section will highlight how changes in glial metabolism may play a role in the physiological response to alterations in energy homeostasis in obesity and diabetes.

### Glia are directly sensitive to changes in nutrient availability

#### Glucose

Astrocytes express glucose transporters (GLUT), most notably GLUT1 and GLUT2 (Morgello et al., 1995, Garcia et al., 2003, Young and McKenzie, 2004), and are critical regulators of brain glucose metabolism (Dienel, 2019). In the hypothalamus, glucose transporter expression is regulated by nutritional state and the hormones leptin and ghrelin (Fuente-Martín et al., 2012, Fuente-Martín et al., 2016), indicating neuroendocrine integration of systemic energy status at the cellular level. Modulation of GLUT2 activity has been implicated in the regulation of food intake and systemic glucose homeostasis in rodents (Marty et al., 2005, Bady et al., 2006, Stolarczyk et al., 2010). Re-expression of GLUT2 in astrocytes of the brainstem is sufficient to restore the counterregulatory response to low-glucose in GLUT2-deficient mice (Marty et al., 2005), indicating the importance of astrocyte glucose sensing in this physiological process. This is further supported by evidence that in astrocytes acute low glucose levels cause structural and functional changes in astrocytes *in vitro* (Lee et al., 2016) and *ex vivo* in brainstem slices (McDougal et al., 2013). Furthermore, work from our group indicates that recurrent exposure to low glucose, mimicking variations often seen in patients with insulin-controlled type-1 diabetes, results in metabolic changes in astrocytes which are likely to be adaptations to preserve brain ATP production in the face of low glucose availability (Weightman Potter et al., 2019). As outlined above, in response to inflammation, on a cellular level astrocytes show compensatory metabolic adaptations to the altered energetic requirements (Yu et al., 1995, Gavillet et al., 2008, Belanger et al., 2011).

Like in astrocytes, emerging evidence supports an intimate relationship between metabolism and inflammatory responses in microglia (Churchward et al., 2018, Nair et al., 2019). Microglia are sensitive to *in vitro* changes in ambient glucose levels which impact their function (Hsieh et al., 2019) and express the glucose transporters GLUT1, GLUT2 and GLUT5 (Payne et al., 1997). Microglia also express ATP-sensitive K^+^ (K_ATP_) channels (Ramonet et al., 2004). Inflammatory stimulation *in vitro* with LPS and interferon-γ results in an adaptation of cellular metabolism in microglia towards glycolysis (Gimeno-Bayon et al., 2014), while inhibition of glycolysis using 2DG attenuates LPS-induced microglial activation (Shen et al., 2017). Together these data suggest that in common with macrophages (O’Neill et al., 2016), glycolytic pathways are important for normal microglial responses to inflammatory stimuli.

#### Lipids

Glia have been implicated in direct fatty acid sensing. In response to ingestion (oral gavage) of a diet rich in this nutrient, saturated fatty acids accumulate in the medial basal hypothalamus of mice (Valdearcos et al., 2014), indicating the potential for a direct action of this nutrient in the brain, particularly at circumventricular sites. *In vitro* in primary cultured cells, inflammatory responses following treatment with saturated fatty acids have been reported in both astrocytes (Gupta et al., 2012) and microglia (Wang et al., 2012, Valdearcos et al., 2014), although when compared head-to-head the inflammatory response of microglia to this stimulus appears to be more pronounced (Valdearcos et al., 2014). Toll-like receptor 4 (TLR4) has been implicated as a receptor mediating the inflammatory effects of fatty acids in cells. Indeed, TLR4 signalling in the hypothalamus regulates systemic energy balance as intracerebroventricular injection of a TLR4-inhibiting antibody, which likely acts in part via receptors found on glia, ameliorates leptin resistance in mice fed HFHS diet (Milanski et al., 2009). However, recent data from macrophages suggests that, while intact TLR4 signalling is required for the inflammatory actions of saturated fatty acids, palmitate is not a direct agonist of this receptor (Lancaster et al., 2018). Instead of direct receptor-mediated activation, it appears that changes in cellular metabolism and membrane lipid composition downstream of TLR4 activation are necessary for the inflammatory effects of saturated fatty acids (Lancaster et al., 2018).

Both microglia and astrocytes express CD36 and loss of this the fatty acid transporter impacts glial function *in vitro* and *in vivo* in response to neurological insult (Bao et al., 2012, Li et al., 2015). This suggests that the ability to take up and utilise fatty acids as an energy source is critical for normal glial function in response to an insult, and is likely related to concomitant immunometabolic responses. In the context of obesity, this phenomenon has been highlighted in the recent work by Gao et al. (2017b) on lipoprotein lipase (LPL) deficiency in microglia. LPL, a key enzyme necessary for the breakdown of triglycerides and uptake of fatty acids, is found in microglia and astrocytes, with higher expression in the former (Gao et al., 2017b). The levels of microglial *lpl* gene expression are increased in diet-induced obese mice and this is associated with alterations in microglial metabolism (Gao et al., 2017a, Gao et al., 2017b). Supporting an important role for microglia in integrating nutritional cues to regulate energy homeostasis, loss of LPL from microglia results in an exacerbation of weight gain (increased adiposity) and a worsening of systemic glucose control in animals fed a HFHS diet (Gao et al., 2017b). This indicates that intact fatty acid uptake by microglia is a key event in normal microglial function in the context of obesity in mice.

#### Amino acids

Branched chain amino acids (BCAAs) can enter the brain through facilitative transport (Smith, 2000) and evidence suggests that the protein composition of the diet influences amino acid concentrations within the hypothalamus (Choi et al., 2001). Several studies indicate that direct infusion of the amino acids leucine or proline into the medial basal hypothalamus influences systemic glucose homeostasis in rodents (Su et al., 2012, Arrieta-Cruz et al., 2013). These effects are mediated via amino acid metabolism in astrocytes which impacts the production of metabolic intermediates by these cells. BCAAs are an important source of glutamate in the brain via the action of BCAA-transaminase (Yudkoff, 2017). Glutamate is a key excitatory neurotransmitter in the brain and glial regulation of its availability and recycling is critical for normal brain function (discussed further below). It is likely that this is achieved via cooperation between neurons and astrocytes and the balance between the activity of the glutamate-glutamine and glutamate-BCAA cycles (Yudkoff, 2017).

### Glial-derived metabolic intermediates and products as signalling molecules

#### ATP

The metabolic endpoint of glycolysis and mitochondrial metabolism results in ATP generation. In most cell types ATP, in addition to being used to fuel biological reactions, is released into the extracellular space (Dosch et al., 2018). Outside cells, extracellular ATP (eATP) acts on purinergic receptors, ionotropic P2X and G-protein coupled P2Y receptors, to increase intracellular calcium and/or activate second messenger signalling (for review see (Jiang et al., 2017)). Release of ATP can occur through connexin/pannexin hemi-channels (Taruno, 2018), vesicular release (Moriyama et al., 2017), lysosomes (Zhang et al., 2007), and by direct membrane damage leading to leakage of intracellular contents. See the review by Franke et al for a comprehensive overview of the pathophysiological role of purinergic signalling in astrocytes, particularly in the regulation of inflammation (Franke et al., 2012). This section will focus on recent developments in neuroendocrine control of purinergic signalling and particularly the control of energy homeostasis.

It is generally accepted that most eATP in the brain comes from astrocytes (Franke et al., 2012). There are a number of P2 receptors expressed in the hypothalamus; for example, orexigenic neuropeptide Y (NPY) and Agouti-Related Protein (AgRP) neurons express the P2X2R (Collden et al., 2010) and steroidogenic factor-1 (SF-1) neurons of the VMH, which also are involved in regulation of feeding, express P2X4, and are excited by ATP (Jo et al., 2011). In the extracellular space ATP can be broken down to adenosine, which acts on adenosine receptors. With relevance to feeding behaviour, chemogenetic activation of hypothalamic astrocytes leads to ATP release and breakdown to adenosine, which inhibits appetite-stimulating AgRP neurons via activation of the adenosine A1 receptor (Yang et al., 2015). It should be noted that in another related study, chemogenetic activation of astrocytes stimulated feeding (Chen et al., 2016), with these differences possibly being a result of subtle differences in experimental design. Recent evidence using astrocyte-specific loss of insulin receptors suggests that insulin stimulates astrocyte ATP release, which is a critical step in the anti-depressive actions of insulin (Cai et al., 2018).

Astrocyte ATP signalling requires a relatively rapid upregulation of glycolysis to enhance pyruvate production, that is subsequently used by the mitochondria to fuel ion handling mechanisms (Juaristi et al., 2019). Whether this enhanced glycolytic response to ATP is driven purely by accelerated glycolysis or is also coupled with enhanced glucose uptake is not clear. In principle however, ATP has been shown to stimulate glucose uptake in other cell types, including skeletal muscle (Osorio-Fuentealba et al., 2013) and kidney cells (Karczewska et al., 2011). In the extracellular space, adenosine is generated from the breakdown of ATP (although can also be a result of direct adenosine release). Activation of adenosine 2B receptors (A2BR) increases glucose uptake into neurons and astrocytes (Lemos et al., 2015). In rodent astrocytes, activation of A1R and A3R attenuates LPS-induced upregulation of glycolysis genes by the transcriptional regulator hypoxia-inducible factor 1α (HIF-1α) (Gessi et al., 2013). These data suggest that in astrocytes, extracellular ATP and its breakdown products may play an important role in regulating glucose uptake and metabolism that influences downstream inflammatory responses. One enzyme that breaks down eATP, CD73, is itself regulated by inflammatory cytokines in cortical astrocytes. For example, LPS and H_2_O_2_ reduce CD73 expression/activity, whilst tumour necrosis factor α (TNF-α) increases expression/activity (Brisevac et al., 2012). This indicates a degree of signal specificity to changes in ATP breakdown extracellularly, plausibly to allow for resolution of inflammation/injury.

#### Lactate

Lactate is released from astrocytes either as a direct product of glycolysis or from the breakdown of small amounts of stored glycogen. In diabetes, specifically in the context of recurrent insulin-induced hypoglycaemia, VMH lactate levels are significantly increased and likely contribute to counterregulatory failure (Borg et al., 2003, Chan et al., 2013). Whether this lactate alters subsequent glial immunometabolic responses relevant to glucose control remains to be determined; however, in separate studies lactate has been demonstrated to influence glial cytokine secretion. For example, in microglial and astrocyte cultures, lactate can induce IL-1β, IL-6 and TNFα secretion from microglia and IL-1β and IL-6 secretion from astrocytes (Andersson et al., 2005). It should be noted that this occurred with high concentrations of lactate (>10 mM) over 8 hours, suggesting this may only happen in relatively extreme pathophysiological contexts. It has also been reported that TNFα and IL-1α can increase astrocyte glucose uptake and utilisation, without an increase in lactate production, suggesting that the additional carbon is being shuttled towards the tricarboxylic acid cycle (TCA) or the pentose phosphate pathway (PPP) (Yu et al., 1995). This is supported by evidence demonstrating a large increase in both TCA and PPP activity in primary mouse astrocytes treated with IL1-β and TNFα with only a modest, non-significant trend toward reduced lactate release (Gavillet et al., 2008). In a Wistar rat neonatal overnutrition model, increased hypothalamic lactate transporter expression, in addition to increases in glucose and glutamate transporter expression (Fuente-Martin et al., 2013) were reported. The addition of sucrose at weaning, subsequent to neonatal overnutrition, also increased serum IL-1β and IL-6 levels and hypothalamic IL-6 mRNA levels (Fuente-Martin et al., 2013). Although these data are not indicative of a role of lactate in neuroinflammation *per se*, when taken together with the data above they suggest that lactate levels are modestly influenced by the inflammatory state, rather than lactate being a driver of the process.

#### Ketone bodies

Ketone bodies are produced in the mitochondria from acetyl-CoA generated primarily from fatty acid oxidation, dependent on the rate-limiting enzymes 3-hydroxy-3-methylglutaryl-CoA (HMG-CoA) synthase and HMG-CoA lyase (Hegardt, 1998, Grabacka et al., 2016). While predominantly synthesised in the liver, astrocytes also produce ketone bodies from fatty acids derived from dietary lipids (Le Foll et al., 2014, Le Foll et al., 2015). While other areas of the brain can also produce ketones, this appears to be enriched in the hypothalamus (Le Foll et al., 2014). In the brain, ketogenesis occurs predominantly in astrocytes which are also the principle oxidisers of fatty acids (Edmond et al., 1987, Bouyakdan et al., 2015, Eraso-Pichot et al., 2018, Sonnay et al., 2019). Both increased dietary fatty acid intake and decreased glucose availability/AMP-activated protein kinase activation can increase astrocytic ketogenesis (Edmond et al., 1987, Blazquez et al., 1999, Le Foll et al., 2014). In fact, more than half of the energy used by neurons during fasting is derived from ketones (Cahill, 2006). Therefore, in both high and low energy states ketogenesis occurs and can act as a signal to regulate food intake. In the context of high fat diets, for example, astrocyte derived ketones can be shuttled to neurons via monocarboxylate transporters where they are metabolised to generate ATP. In this way, they could override normal fatty acids sensing by CD36 via closing K_ATP_ channels and depolarising the cell, thus activating fatty acid-sensing neurons (Le Foll and Levin, 2016). The activation of these fatty acid-sensing neurons by ketones suppresses food intake in both acute HFHS diet fed and diet-induced obese rodent models (Le Foll and Levin, 2016). This is supported by the finding that inhibition of VMH ketone production in rats increases food intake (Le Foll et al., 2015).

In addition to promoting weight loss, ketogenic diets are reported to improve symptoms in several neurological disorders (Yang et al., 2019), including epilepsy (Rho et al., 2019), Alzheimer’s disease (Reger et al., 2004), Parkinson’s disease (Vanitallie et al., 2005), and traumatic brain injuries (White and Venkatesh, 2011). The protective effects of ketones are thought to be mediated in part by immune-modulatory actions. For example, β-hydroxybutyrate blocks the NLR Family Pyrin Domain Containing 3 (NLRP3) inflammasome (Youm et al., 2015), which mediates the release of pro-inflammatory cytokines IL-1β and IL-18 in brain (Yatsiv et al., 2002, de Rivero Vaccari et al., 2009, Li et al., 2011). It is hypothesised that ketone-induced inhibition of NLRP3 suppresses innate immune responses during starvation to spare ATP generated for maintenance of cell function (Youm et al., 2015). Interestingly, NLRP3 contributes to the onset of type 2 diabetes by increasing insulin resistance, which can be attenuated by ablation of NLRP3 (Vandanmagsar et al., 2011, Wen et al., 2011).

#### Glutamate/glutamine

Astrocytes recycle glutamate from the synapse to regulate neurotransmission and prevent excitotoxicity (Verkhratsky and Nedergaard, 2018). Briefly, astrocytes sequester glutamate from the synapse via GLT-1/EAAT2 or glutamate aspartate transporter (GLAST/EAAT1). Glutamate is then converted to glutamine by glutamine synthase where it is either further metabolised by astrocytes via the TCA cycle or transported back to neurons via system-N amino acid transporters (SNATs). Once glutamine has re-entered the neuron, phosphate activated glutaminase (PAG) converts it to glutamate where it can be repackaged into vesicles in preparation for neurotransmitter release. Microglia also take up glutamate via GLT-1/EAAT2 where it is converted to glutathione which is an important defence against reactive oxygen species (ROS) (Persson et al., 2006). The uptake of glutamate by astrocytes and microglia is metabolically expensive, requiring activity of the Na^+^/K^+^-ATPase. To satisfy this increased energetic demand, evidence suggests that astrocytes increase glycolysis and lactate production within seconds of exposure to glutamate (Pellerin and Magistretti, 1994).

In states of high neuronal activity or damage, excessive demands on astrocytes to sequester neurotransmitters like glutamate can negatively impact their ability to do so. For example, in rats normal hypoglycaemia-induced glutamate release in the VMH is suppressed after repeated-hypoglycaemia, attributed in part to a decrease in astrocyte glutamate uptake (Chowdhury et al., 2017). Therefore, the normal glutamatergic response to a decrease in glucose is attenuated, delaying the onset of the normal counterregulatory response to hypoglycaemia (Chowdhury et al., 2017). On the other end of the energy homeostasis spectrum, intake of an obesogenic HFHS diet rapidly increases hypothalamic glutamatergic signalling (Guyenet et al., 2013) and expression of astrocytic glutamate transporter expression (Fuente-Martín et al., 2012). Furthermore, chronic intake of a HFHS diet alters microglial expression of genes associated with glutamate metabolism such that the normal antioxidant effects of glutamate-derived glutathione are suppressed, increasing ROS production (Milanova et al., 2019). It is possible that chronically elevated glutamatergic signalling associated with diet-induced obesity increases the metabolic demand on both microglia and astrocytes to prevent excitotoxicity. This in turn negatively impacts their ability to support neuronal activity; thus, contributing to hypothalamic synaptic dysfunction and the death of ARC POMC neurons (Horvath et al., 2010, Thaler et al., 2012, Reis et al., 2015).

#### Reactive oxygen species

Reactive oxygen species (ROS) are a by-product of mitochondrial activity and at high levels can cause oxidative stress in the cell. Despite the negative impact of excess ROS on cellular stress, ROS production by astrocytes may have a role in regulating normal CNS function (Vicente-Gutierrez et al., 2019). Astrocytes cultured from mice with astrocyte-specific down-regulation of ROS production had altered cellular metabolism: glycolysis was reduced while activity of the PPP increased, and a lower NADPH(H^+^)/NADP^+^ ratio was observed. *In vivo,* mice with reduced astrocytic ROS production displayed altered neuronal dendrite projections in the hippocampus and parietal cortex, alongside cognitive impairment (Vicente-Gutierrez et al., 2019). While accumulation of ROS can be pathological when dysregulated, this aforementioned study and others (Angelova and Abramov, 2016) implicate astrocytic ROS production as a part of normal physiological regulation of cellular signalling pathways.

## Future perspectives

**Fig. 1:**
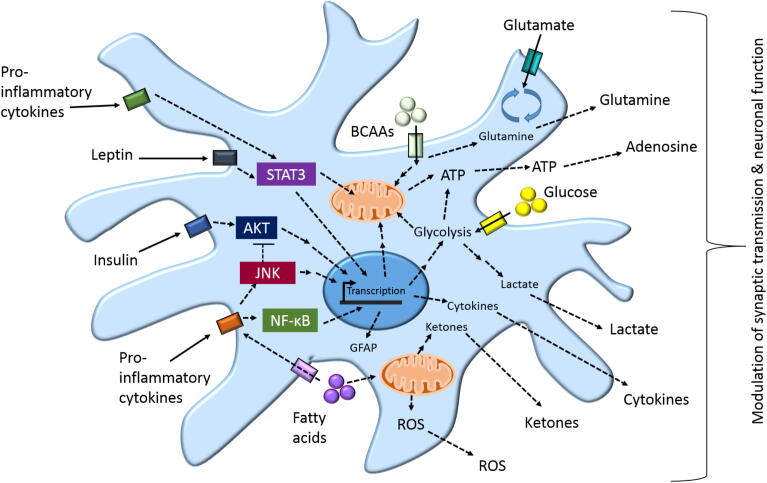
A simplified schematic diagram of the proposed immunometabolic changes in astrocytes which may contribute to the CNS regulation of energy homeostasis – A variety of inputs, including nutritional stimuli (glucose, fatty acids, and branch chain amino acids [BCAA]), hormones (leptin and insulin) and cytokines can directly regulate cellular signalling and metabolic pathways in astrocytes. The signalling pathways implicated include signal transducer and activator of transcription 3 (STAT3), AKT, c-Jun N-terminal kinase (JNK) and nuclear factor kappa B (NF-κB), which in turn can impact astrocyte metabolism by modulating transcription or translation of key regulatory components of glycolysis and mitochondrial metabolism. Depending on the signalling pathway(s) activated and the energetic status of the cell, which is directly impacted by the CNS microenvironment, this leads to changes in levels of metabolic intermediates and products: lactate, ketones, reactive oxygen species (ROS), glutamine, and ATP. When released from the cell, these factors are capable of modulating synaptic transmission and function of neurons in energy homeostasis circuits in the CNS. Activation of astrocyte signalling can also lead to the production of cytokines, which may act in an autocrine or paracrine fashion to modulate neuronal and glial activity, potentially perpetuating a CNS proinflammatory microenvironment, such as in obesity or diabetes. In addition to modulating release of biochemical signalling modules, activation of astrocyte signalling can lead to changes in expression of structural proteins in astrocytes, such as glial-fibrillary acidic protein (GFAP), which enable morphological changes in astrocytes which impact neuronal transmission via ensheathment of synapses.

In common with peripheral immune cells, accumulating evidence suggests that immunometabolic changes in glia may be instrumental in the pathophysiology of obesity and diabetes. Additional work is required to understand the full spectrum of these responses, particularly with respect to the molecular mechanisms by which hormones and nutrients impact glial function, and how these glial changes in turn impact synaptic transmission and neuronal function. In this review we have focused on highlighting some of the key signalling pathways and metabolic processes that may be involved (see Fig. 1 for a summary of astrocytic changes, many of which are also applicable to microglia). Much of our current understanding of immunometabolic responses in glia comes from *in vitro studies* on isolated cell types in culture which, while informative, cannot fully recapitulate the complex interactions between glia and neurons in the *in vivo* environment. Studies examining mice with genetic modification of glial function have begun to reveal a key role for these cells in modulating neuronal circuitry regulating energy homeostasis; however, compared with neurons, the available tools for specific inducible-modulation of glial cell function are limited which is currently restricting the ability to elucidate the spatial–temporal dynamics of glial modulation of neural circuits. Another challenge in the field is the fact that we still know little about regional heterogeneity in glial function, both generally and with respect to their role in modulating neural circuits controlling energy homeostasis. To date most of the work on glial modulation of CNS circuits regulating energy homeostasis has focused on the hypothalamus, with other key areas such as the dorsal-vagal complex of the brainstem and mesolimbic reward pathways being comparatively understudied. In the context of obesity and diabetes an improved understanding of how (and where) changes in glial function are initiated and regulated is needed, before we can consider developing therapeutic interventions targeting this system. This should be complemented by continued efforts to understand how glia contribute to the regulation of systemic energy homeostasis in the course of normal physiology.
